# Immunomodulatory Effects of *Periplaneta americana* Oligosaccharides Through SCFA-Producing Gut Microbiota and Metabolic Regulation in Immunosuppressed Mice

**DOI:** 10.3390/biom16040496

**Published:** 2026-03-25

**Authors:** Kaimin Lu, Chunyan Zhang, Jinku Bao

**Affiliations:** 1 Pharmacy Research Center, Binzhou Medical University, Yantai 264003, China; 2 Key Laboratory of Bio-Resource and Eco-Environment of Ministry of Education, College of Life Sciences, Sichuan University, Chengdu 610065, China; chunyanzhang@sntcm.edu.cn; 3 School of Basic Medicine, Shaanxi University of Chinese Medicine, Xianyang 712046, China

**Keywords:** oligosaccharides, SCFA, gut microbiota, immunosuppression, metabolomic

## Abstract

Immunosuppression is associated with impaired immune responses and increased susceptibility to disease, highlighting the need for safe and effective immunomodulatory strategies. Oligosaccharides derived from natural sources have attracted growing interest due to their bioactivity and regulatory effects on host immunity. The present study was designed to evaluate the immune-enhancing potential of *Periplaneta americana* oligosaccharides (PAOSs) and to explore their association with SCFA-producing gut microbiota and metabolic regulation in an immunosuppressed mouse model. PAOS administration significantly increased serum immunoglobulin levels (IgG and IgM), promoted the secretion of immunoregulatory cytokines (IFN-γ, IL-2, TNF-α, IL-10, and IL-4), and elevated the proportion of CD4^+^ T cells in the spleen. In addition, PAOSs alleviated oxidative stress by reducing malondialdehyde accumulation while promoting the activity of key antioxidant enzymes, such as superoxide dismutase, catalase, and glutathione peroxidase. Metabolomic analysis revealed that PAOSs altered host metabolic profiles, particularly enhancing pyrimidine metabolism. Furthermore, PAOSs markedly enriched short-chain fatty acid (SCFA)-producing bacteria, and elevated colonic short-chain fatty acid levels. These changes were closely associated with the observed improvement in immune function. Collectively, this study demonstrated that PAOSs exerted immunomodulatory effects through coordinated regulation of SCFA-producing gut microbiota and host metabolism, elucidating the mechanisms underlying the bioactivity of insect-derived oligosaccharides.

## 1. Introduction

The immune system functions as a fundamental defensive system that safeguards the organism against exogenous pathogens, and it plays a crucial role in preserving host health and physiological integrity [1,2]. Immunosuppression is a pathological condition marked by the attenuation of immune responses, rendering the host increasingly vulnerable to bacterial or viral infections, tumor progression, and impaired tissue repair [3,4,5]. It may arise secondary to chemotherapeutic regimens, organ transplantation, autoimmune disorders, or chronic stress. Cyclophosphamide (CTX) is widely employed in cancer clinical chemotherapy [6], but its use is often accompanied by immunosuppression and various toxic side effects which can severely compromise the immune system [7,8]. Since enhancing immune function is an effective strategy to reduce chemotherapy-induced immunological dysfunction and disease incidence, several immunomodulators have been widely utilized [7,9,10]. However, current clinical immunopotentiators, for example levamisole and Isoprinosine, are associated with undesirable adverse reactions, including headaches, fatigue, fever, neutropenia, leukopenia, and hypersensitivity responses [11,12,13]. Hence, it is meaningful to the development of safe and effective immunomodulators to ameliorate immunosuppression.

As conventional immunostimulatory therapies often come with undesirable side effects or limited efficacy, there is increasing interest in natural immunomodulators with improved biocompatibility and safety profiles [13,14]. Numerous natural products, such as polysaccharides or oligosaccharides, possess immunomodulatory properties while exhibiting minimal or no toxicity to the host [15,16,17]. Thus, the identification and development of natural immunomodulators—such as bioactive oligosaccharides—has garnered increasing attention in the contemporary biomedical research field.

Previous studies have demonstrated that oligosaccharides derived from natural sources can enhance immune responses by regulating cytokine production, antioxidant capacity, and immune cell activity [18]. Among immune cell populations, CD4^+^ T cells play a central role in orchestrating adaptive immune responses by regulating cytokine secretion and coordinating the activity of other immune cells. CD4^+^ T cells are an important population of adaptive immune cells, and changes in their proportion are closely associated with immune status and host defense capacity [19]. Therefore, the modulation of CD4^+^ T cell responses has become an important mechanism through which many natural bioactive compounds exert immunomodulatory effects.

Naturally derived oligosaccharides exert potent immunomodulatory effects by interacting with pattern recognition receptors [20,21], thereby activating both innate and adaptive immune responses. *Periplaneta americana*, a traditional Chinese medicinal insect, has a 300-million-year evolutionary history and is broadly distributed across tropical zones [22,23]. *Periplaneta americana* is widely used in the treatment of infant malnutrition, tonsillitis, phlegm retention, carbuncles, and sore throat. It also exhibits immunomodulatory, mucosal repair, and antitumor effects, and can act as antibacterial, antiviral, analgesic, and antioxidant agents. *Periplaneta americana* contains various bioactive components, including peptides, polysaccharides, glycoproteins, and oligosaccharides, which contribute to its pharmacological activities [22,23]. Oligosaccharides derived from *Periplaneta americana* have recently attracted increasing attention because of their potential biological activities. In our earlier work, it was demonstrated that *Periplaneta americana* oligosaccharides (PAOSs) can enhance immune function by regulating immune responses, attenuating oxidative stress, and reshaping intestinal flora with high safety in diabetic mice [24,25]. Nevertheless, the exact mechanisms by which PAOSs exert immunomodulatory effects, especially under immunosuppressed conditions, have yet to be fully elucidated. Therefore, this study aims to investigate the potential of PAOSs in reversing chemically induced immunosuppression in vivo. To comprehensively evaluate the immunomodulatory effects of PAOSs, multiple immune-related parameters were assessed in this study. Cytokines and immunoglobulins are important signaling molecules that regulate immune cell communication and inflammatory responses. Oxidative stress is closely associated with immune function, as excessive reactive oxygen species can impair immune cell activity and promote inflammatory processes [26]. In addition, the composition of immune cell populations reflects the functional state of the immune system. Emerging evidence suggests that metabolic reprogramming plays an important role in immune regulation; therefore, serum metabolomic profiling can provide insights into the metabolic mechanisms underlying immune responses [27]. Furthermore, short-chain fatty acids (SCFAs), major metabolites produced by gut microbiota, have been shown to regulate immune responses by influencing cytokine production and immune cell differentiation [28]. By systematically assessing the effects of insect-derived oligosaccharides on immunoglobulin and cytokine production, oxidative stress, immune cell populations, serum metabolic profiles, and gut microbiota–SCFAs, the present study highlights the immunomodulatory potential of insect-derived oligosaccharides in the context of immune-related disorders.

## 2. Materials and Methods

### 2.1. Preparation of PAOS

The PAOS was acquired following the experimental procedures we established in earlier studies [24]. The *Periplaneta americana* residues were supplied by the Sichuan Gooddoctor-Panxi Pharmaceutical Company (Xichang, Sichuan, China). The PAOS extraction was performed with the assistance of a monomode microwave-assisted synthesis machine (Monowave 300, Anton Paar, Graz, Austria), in which the sample was treated with phosphate-buffered saline (PBS, pH 6.5) at a weight-to-weight ratio of 1 to 10 under 560 watts for 1 min. Subsequently, the mixture was enzymatically hydrolyzed with 10,000 U of papain (Solarbio, Beijing, China) at 65 °C over a 4 h incubation period. The extract was then subjected to size-exclusion chromatography using a Sephacryl S-100 high-resolution column (1.5 × 95 cm, GE, Boston, MA, USA). The third elution peak was collected, concentrated, and lyophilized for further analysis. PAOS was composed of glucose, galactose, and xylose, and the backbone was (1→4)-Glcp with a molecular mass of 1.0 kDa [24].

### 2.2. Animals and Study Design

Male Kunming mice aged 5 weeks were obtained from Charles River Laboratory Animal Technology Co., Ltd. (Beijing, China; License No. SCXK (Jing) 2016-0011). Prior to experimentation, all animals were acclimatized for one week under controlled conditions (temperature: 22 ± 2 °C; relative humidity: 50 ± 5%; 12 h light/dark cycle) with ad libitum access to standard chow and water. All animal experiments were conducted following the guidelines of the Animal Care and Use Committee of China. The present research was in full compliance with the NIH Guide for the Care and Use of Laboratory Animals and approved by Research Ethics Committee of Binzhou Medical University (Approval No. 2021-310, Approval Date: 29 November 2021).

Subsequent to acclimatization, the test mice were randomly assigned into six distinct experimental groups (n = 14 per group): normal group (N, PBS), CTX model group (M, PBS), low-dose PAOS group (L, 100 mg/kg), high-dose PAOS group (H, 300 mg/kg) and positive group (P, levamisole hydrochloride, 10 mg/kg, MCE, Monmouth Junction, NJ, USA). Mice were housed at 3–4 mice per cage (four cages per group). Among the animals in each group, six mice were designated for the ear swelling assay, while the remaining mice were used for other analyses. Mice were randomly assigned to groups via random drawing, which was conducted by an independent researcher. The immunosuppressed mouse model was established via intraperitoneal administration of 80 mg/kg CTX (MCE, Monmouth Junction, NJ, USA) for three consecutive days [29].

All interventions were delivered via oral gavage on a daily basis over a 15-day continuous period. Body weight was monitored throughout whole animal experiments. Relative body weight was calculated as follows:

Relative body weight (%) = (day n body weight/day 1 body weight) × 100%(1)

**Figure 1:**
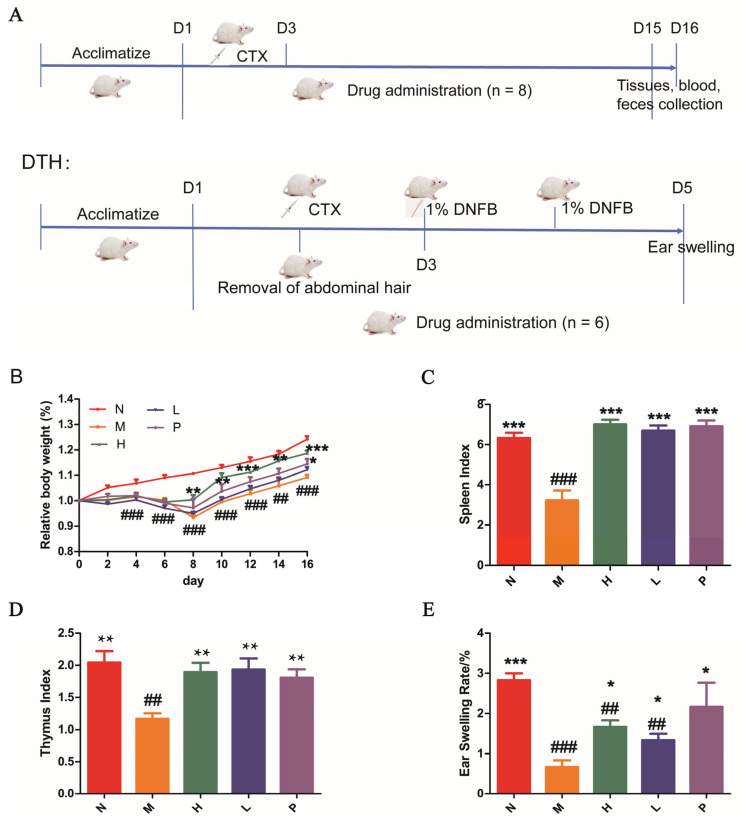
PAOSs alleviated the immunosuppressed symptoms in CTX-induced immunosuppression. (**A**) Schematic overview of the experimental design; (**B**) the changes in relative body weight during the experiment; (**C**) spleen index; (**D**) thymus index, data are expressed as the mean ± SD (n = 8); (**E**) ear swelling rate, data are expressed as the mean ± SD (n = 6). N: normal group; M: immunosuppressed model group; H: DM + high-dose PAOS group; L: DM + low-dose PAOS group; P: DM + levamisole hydrochloride group. *** *p* < 0.001, ** *p* < 0.01, * *p* < 0.05 vs. M group; ^###^ *p* < 0.001, ^##^ *p* < 0.01 vs. N group.

Blood samples were immediately collected via cardiac puncture under deep anesthesia. When the experimental procedure ended, all mice were euthanized through exposure to carbon dioxide. A schematic diagram of the experimental design is presented in Figure 1A.

### 2.3. Thymus and Spleen Index

The thymus and spleen were carefully dissected and weighed. The organ indices were determined using the formula presented below:

Spleen index (mg/g) = Spleen weight (mg)/Body weight (g)(2)

Thymus index (mg/g) = Thymus weight (mg)/Body weight (g)(3)

### 2.4. Ear Swelling Assay

Six mice in each group were randomly designated to conduct the DTH test. On the second day of gavage administration, the abdominal hair of all mice was removed using depilatory cream prior to sensitization. On the third day, 50 μL of 1% DNFB (2,4-dinitrofluorobenzene) was applied to the shaved abdominal area for initial sensitization. On the fourth day, a secondary sensitization was performed by applying 10 μL of 1% DNFB to the right auricle as a challenge. After 24 h, the mice were euthanized, and ear swelling was assessed by measuring the thickness of bilateral auricles using a micrometer.

### 2.5. Determination of Immunoglobulins and Cytokines in Serum

The collected blood specimens were kept stationary at ambient temperature for 30 min and subsequently centrifuged at 2000× *g* for 15 min to obtain the serum. The levels of serum immunoglobulins (IgA, IgG, and IgM), as well as cytokines (INF-γ, IL-2, TNF-α, IL-10, and IL-4) were measured with ELISA kits (Mlbio, Shanghai, China), in strict accordance with the vendor’s protocols.

### 2.6. Measurement of Oxidative Stress

Liver and kidney tissues were subjected to homogenization, followed by the measurement of protein concentration using a BCA protein assay kit (NCM, Suzhou, China). The levels of superoxide dismutase (SOD), malondialdehyde (MDA), catalase (CAT), and glutathione peroxidase (GSH-Px) were detected using ELISA kits (Mlbio, Shanghai, China).

### 2.7. Morphological Analysis of Spleen

Splenic samples were immobilized in 4% paraformaldehyde over one full day. The specimens were then paraffin-embedded, sliced to a thickness of 2–4 μm, deparaffinized with xylene, and dehydrated via graded ethanol. Lastly, the tissue slices underwent hematoxylin and eosin (H&E) and visualized under an optical microscope. Imaging was performed using a Nikon Eclipse Ci optical microscope equipped with a Nikon DS-U3 imaging system (Nikon Corporation, Tokyo, Japan).

Histopathological evaluation of spleen tissues was performed based on a semi-quantitative scoring system. The scoring criteria included three parameters: white pulp integrity, red pulp expansion, and inflammatory cell infiltration [30]. Each parameter was scored on a scale of 0–4 according to the severity of histological alterations (Table 1), and the final score for each sample was calculated as the sum of the three parameters (total score: 0–12).

### 2.8. Immunofluorescence Analysis of CD4^+^ and CD8^+^ T Cells in Spleen Tissue

Spleen tissues were immobilized with 4% paraformaldehyde, dehydrated, embedded in paraffin, and sliced. The specimens were incubated using 10% normal goat serum for 1 h. Subsequently, primary antibodies targeting mouse CD4 and CD8 antigens (BD Biosciences, Franklin Lakes, NJ, USA) were incubated, followed by overnight incubation of the sections at 4 °C. Subsequent to rinsing with PBS, fluorescently conjugated secondary antibodies were supplemented and incubated for 1 h at ambient temperature under light-proof conditions. The sections were then flushed with PBS and counterstained using DAPI for 5 min, followed by another PBS wash. Ultimately, the sections were coverslipped using an anti-fade mounting reagent and visualized via a fluorescence microscope (Leica, Wetzlar, Germany) to detect CD4^+^ and CD8^+^ cells.

### 2.9. Flow Cytometry Assay

The spleen was collected and ground in PBS, followed by filtration through a 200-mesh nylon cell strainer to prepare single-cell suspensions. With regard to the assessment of T lymphocyte subpopulations, the splenocyte suspensions were stained on ice for half an hour using the following fluorochrome-conjugated monoclonal antibodies: FITC-anti-mouse CD3, APC-anti-mouse CD8, and PE-anti-mouse CD4 (Elabscience, Wuhan, China). The percentages of T cell subsets were analyzed using a BD flow cytometer (BD, Franklin Lakes, NJ, USA). A total of 10,000 target events were gathered in list mode, and the fluorescence staining results were analyzed using FlowJo CE software (version 10.8.1, Ashland, OR, USA).

### 2.10. Non-Targeted Metabolomics Analysis

An LC ultra-high performance liquid chromatography (UHPLC, Agilent, Santa Clara, CA, USA) system coupled with a Triple TOF 6600 mass spectrometer (AB, SCIEX, Framingham, MA, USA) was used for raw data acquisition of serum metabolomics. Serum specimens were blended with pre-chilled methanol/acetonitrile/water solution (2:2:1, *v*/*v*), vortexed, and ultrasonicated at 4 °C for 30 min. The mixture was incubated at −20 °C for 10 min, then centrifuged at 14,000× *g* for 20 min under 4 °C conditions. The supernatant was collected and lyophilized. For mass spectrometry analysis, 100 μL of acetonitrile/water solution (acetonitrile: water = 1:1, *v*/*v*) was added to reconstitute the sample, which was then vortexed and centrifuged at 14,000× *g* and 4 °C for 15 min. The resulting supernatants were separated using an Agilent 1290 Infinity LC UHPLC system with a HILIC column. The column temperature was set at 25 °C, the flow rate at 0.5 mL/min, and the injection volume at 2 μL. The mobile phase consisted of: phase A: water + 25 mM ammonium acetate + 25 mM ammonia water; phase B: acetonitrile. The gradient elution program was as follows: 0–0.5 min: 95% B; 0.5–7 min: B linearly decreased from 95% to 65%; 7–8 min: B linearly decreased from 65% to 40%; 8–9 min: B maintained at 40%; 9–9.1 min: B linearly increased from 40% to 95%; 9.1–12 min: B maintained at 95%. To avoid interference from fluctuations in instrument detection signals, samples were analyzed consecutively in a random order. Quality control reference specimens were interspersed in the sample sequence to verify and evaluate the robustness of the detection system and the credibility of the generated experimental data.

After separation by the Agilent 1290 Infinity LC UHPLC system, samples were analyzed by the AB Triple TOF 6600 mass spectrometer using electrospray ionization (ESI) in both positive and negative ion modes. The ESI source parameters were set as follows: Gas1 (nebulizer auxiliary heating gas): 60; Gas2 (auxiliary heating gas): 60; curtain gas (CUR): 30 psi; ion source temperature: 600 °C; ion spray voltage (ISVF): ±5500 V (for both ion modes). Detection ranges and acquisition parameters were set as follows: primary mass-to-charge ratio (m/z) range: 60–1000 Da; secondary product ion m/z range: 25–1000 Da; primary mass spectrum scan accumulation time: 0.20 s/spectra; secondary mass spectrum scan accumulation time: 0.05 s/spectra. Declustering potential (DP) was ±60 V (for both ion modes), and collision energy was 35 ± 15 eV.

Primary spectral data were initially converted to mzXML files via ProteoWizard, further processed with XCMS software (version 3.16.1) for peak matching, retention time calibration, and peak area derivation. Raw data were preprocessed by removing metabolites with missing values in more than 50% of samples, and the remaining missing values were imputed using the k-nearest neighbors method. Data were normalized using sum normalization and subsequently scaled by Pareto scaling prior to statistical analysis. Principal component analysis (PCA) was first conducted as an unsupervised approach to evaluate overall data quality, sample distribution, and potential outliers. Orthogonal partial least squares-discriminant analysis (OPLS-DA) was then performed to identify metabolic differences between groups. Model robustness was assessed using 200-times permutation tests to avoid overfitting. Quality control samples were analyzed intermittently throughout the run to monitor instrument stability, and signal drift was corrected using QC-based support vector regression. Metabolite identification was achieved based on accurate mass matching (mass error < 25 ppm), MS/MS spectral similarity (score > 0.7), and retention time, with annotation confidence reaching at least Level 2. Differential metabolites were identified based on VIP > 1 and *p* < 0.05 for pairwise comparisons, and one-way ANOVA (*p* < 0.05) for multiple group comparisons. Hierarchical clustering and correlation analyses were then performed on the potential biomarkers. The pathway enrichment analysis was performed by KEGG database (https://www.kegg.jp/, accessed on 15 January 2025) and MetaboAnalyst (https://www.metaboanalyst.ca/, accessed on 18 January 2025).

The data reported in this paper have been deposited in the OMIX, China National Center for Bioinformation/Beijing Institute of Genomics, Chinese Academy of Sciences (https://ngdc.cncb.ac.cn/omix: accessed on 10 March 2025, accession no. OMIX 015597).

### 2.11. Metagenomic Sequencing and Analysis of SCFA-Related Microbial Features

Fecal samples were collected under sterile conditions and immediately stored at −80 °C until analysis. Microbial genomic DNA was extracted using a commercial DNA isolation kit following the manufacturer’s protocol. DNA integrity and concentration were assessed prior to library preparation. Shotgun metagenomic libraries were constructed and sequenced on an Illumina platform to generate paired-end reads.

Metagenomic sequencing data were preprocessed using fastp (version 0.20.0) with default parameters to obtain high-quality clean reads by removing adapter-contaminated reads, reads containing more than 10% ambiguous bases, and reads with over 50% low-quality bases (Q ≤ 15). For host-associated samples, potential host DNA contamination was removed by aligning reads to the host reference genome using BWA (version 0.7.9a), and mapped reads were discarded. The remaining high-quality reads were assembled de novo using MEGAHIT (version 1.1.2) based on succinct de Bruijn graphs, and contigs ≥ 500 bp were retained for downstream analysis. Open reading frames were predicted from assembled contigs using Prodigal, and sequences with a minimum length of 200 bp were translated into amino acid sequences. A non-redundant gene catalog was constructed by clustering predicted genes using CD-HIT (version 4.6.1) at 95% sequence identity and 90% coverage, with the longest sequence in each cluster selected as the representative. Finally, gene abundance was calculated by aligning clean reads to the non-redundant gene set using Bowtie2, and abundance profiles were generated based on mapping results.

### 2.12. Analysis of SCFAs

A total of 200 mg of mouse feces was resuspended in 50 μL of 20% phosphoric acid. Afterwards, an isopropyl ether solution containing 500 μM internal standard was added, and the mixture was vortexed thoroughly. Subsequently, the blended solution was processed via centrifugation at 14,000× *g* and at 4 °C for 20 min. Gathered supernatant fraction was transferred to a sample vial, which was then subjected to GC-MS analysis. Samples were separated using an Agilent DB-FFAP capillary (Agilent, Santa Clara, CA, USA) column (30 m × 250 μm × 0.25 μm) coupled with a GC system. For MS analysis, an Agilent 5977B MSD mass spectrometer (Agilent, Santa Clara, CA, USA) was employed. Peak areas and retention times of the chromatograms were obtained via the MSD ChemStation software (version E.02.02). Calibration curves were plotted to quantify the content of SCFAs in the test specimens.

The data reported in this paper have been deposited in the OMIX, China National Center for Bioinformation/Beijing Institute of Genomics, Chinese Academy of Sciences (https://ngdc.cncb.ac.cn/omix: accessed on 10 March 2025, accession no. OMIX 015601).

### 2.13. Statistical Analysis

Data are expressed as mean ± standard deviation (SD). Differences among groups were compared using one-way analysis of variance (ANOVA) followed by Tukey’s multiple comparison test performed with GraphPad Prism 5 software. For body weight measurements collected over time, two-way ANOVA was performed to evaluate the effects of treatment and time, followed by appropriate multiple comparison tests. Prior to analysis, data distribution normality and homogeneity of variance were assessed using the Shapiro–Wilk test and Levene’s test, respectively. Multiple comparisons were corrected as appropriate within the ANOVA framework. A *p*-value of less than 0.05 was considered statistically significant, while a *p*-value of less than 0.01 was regarded as highly significant.

## 3. Results

### 3.1. PAOSs Alleviated the Immunosuppressed Symptoms

CTX-treated mice showed a substantially lower body weight than those in the normal group, and the relative body weight was markedly improved in the positive group and PAOS treatment group (Figure 1B), which suggested that PAOSs could suppress the weight loss in immunosuppressed mice.

The thymus and spleen, as important immune tissues of the body, serve as indicators of the immune status of the entire body. The thymus index and spleen index act as intuitive indicators of the innate immune function of the body. As shown in Figure 1C,D, compared with the normal group, the organ indices of the spleen and thymus in the model group were significantly decreased. Compared to the model group, the spleen index (*p* < 0.001) and thymus index (H, *p* = 0.0017; L, *p* = 0.0026; P, *p* = 0.0024) of PAOS high/low-dose group and positive group mice markedly increased and returned to the normal level. These indicated that PAOSs enhanced immunity by increasing the non-specific immune level in immunosuppressed mice.

The DTH reaction induced by DNFB is a pathological response mediated by Th1 cells. As a vital cell-mediated pathological reaction, it exerts a pivotal function in assessing T cell-mediated immune activities. As seen in Figure 1E, the ear swelling degree in the model group was considerably reduced in comparison with the normal group (*p* < 0.001). After PAOS treatment, the ear swelling degree was distinctly elevated compared to that of the model group (H, *p* = 0.0132; L, *p* = 0.0447), which indicated that PAOSs promoted T cell-mediated specific immune responses to enhance immune activity.

### 3.2. PAOSs Benefited Immunoglobulins and Cytokine Parameters in Immunosuppressed Mice

**Figure 2:**
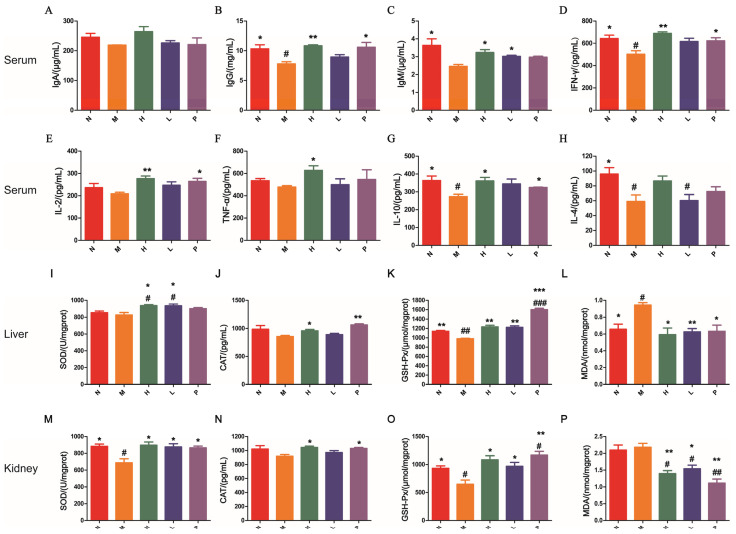
PAOSs benefited immunoglobulins and cytokine parameters, and suppressed oxidative stress parameters of the liver and kidney in immunosuppressed mice. Levels of IgA (**A**), IgG (**B**), IgM (**C**), IFN-γ (**D**), IL-2 (**E**), TNF-α (**F**), IL-10 (**G**) and IL-4 (**H**) in serum. Levels of SOD (**I**), CAT (**J**), GSH-Px (**K**) and MDA (**L**) in the liver, and levels of SOD (**M**), CAT (**N**), GSH-Px (**O**) and MDA (**P**) in the kidney. Data are expressed as the mean ± SD (n = 6). N: normal group; M: immunosuppressed model group; H: DM + high-dose PAOS group; L: DM + low-dose PAOS group; P: DM + levamisole hydrochloride group. *** *p* < 0.001,** *p* < 0.01, * *p* < 0.05 vs. M group; ^###^ *p* < 0.001, ^##^ *p* < 0.01, ^#^ *p* < 0.05 vs. N group.

The immunomodulatory effects of PAOSs on mice were assessed by measuring the levels of immunoglobulins and cytokines in serum. Immunoglobulins constitute a vital element of the body’s immune system and exert a key function in maintaining the organism’s immune function. IgA content in the model group exhibited no statistically significant difference in comparison with the normal group (Figure 2A, *p* > 0.05). The contents of IgG and IgM in the model group were notably reduced by 24.6% and 32.2% than those of the normal mice (Figure 2B,C, *p* = 0.0313, *p* = 0.0376). Meanwhile, the levels of IgG and IgM in the high-dose PAOS treatment group were elevated by 39.3% and 31.7%, and in the positive group they were significantly increased by 36.1% and 20.7%, which returned to normal levels. Cytokines are signaling molecules which can directly or indirectly regulate immune functions. The contents of serum cytokines are illustrated in Figure 2D–H. Compared with the normal group, the level of IFN-γ, IL-10 and IL-4 were significantly decreased by 21.9%, 24.9% and 38.5% in the model group, respectively (*p* = 0.0303. *p* = 0.0337, and *p* = 0.0398). Compared with the model group, the level of IFN-γ, IL-2, TNF-α, IL-10 and IL-4 in the high-dose PAOS treatment group were significantly elevated by 37.1%, 32.5%, 31.1%, 32.5%, and 46.9%, respectively. These findings reveled that PAOSs boosted the immune status of immunosuppressed mice by stimulating the secretion of immunoglobulins and cytokines.

### 3.3. PAOSs Suppressed Oxidative Stress

Oxidative damage is caused by the generation of free radicals and the loss of antioxidant defense, resulting in excessive ROS, and is associated with abnormal immune responses. We investigated the impacts of PAOSs on the oxidative stress status in the liver and kidney. As illustrated in Figure 2I–K, compared with model mice, the levels of antioxidant enzymes including SOD, CAT, and GSH-Px were higher by 13.4%, 12.2% and 25.9% in liver tissues in the high-dose PAOS treatment group, respectively. Consistent with the above results, in kidney tissues, the levels of SOD, CAT, and GSH-Px were higher by 30.6%, 13.5%, and 32.1% in the high-dose PAOS treatment group, and were higher by 27.6%, 5.7%, and 20.8% in the low-dose PAOS treatment group than those in the model group (Figure 2M–O). On the contrary, as revealed in Figure 2L,P, the levels of MDA (maker of oxidative stress) in liver and kidney tissues of the PAOS treatment group or positive group were notably reduced (liver: H, *p* = 0.0133; L, *p* = 0.0027; P, *p* = 0.0168; kidney: H, *p* = 0.0059; L, *p* = 0.0159; P, *p* = 0.0032). Collectively, these results demonstrated that PAOSs had potential efficacy in reducing oxidative stress.

### 3.4. PAOSs Ameliorated Pathological Injury in the Spleen

**Figure 3:**
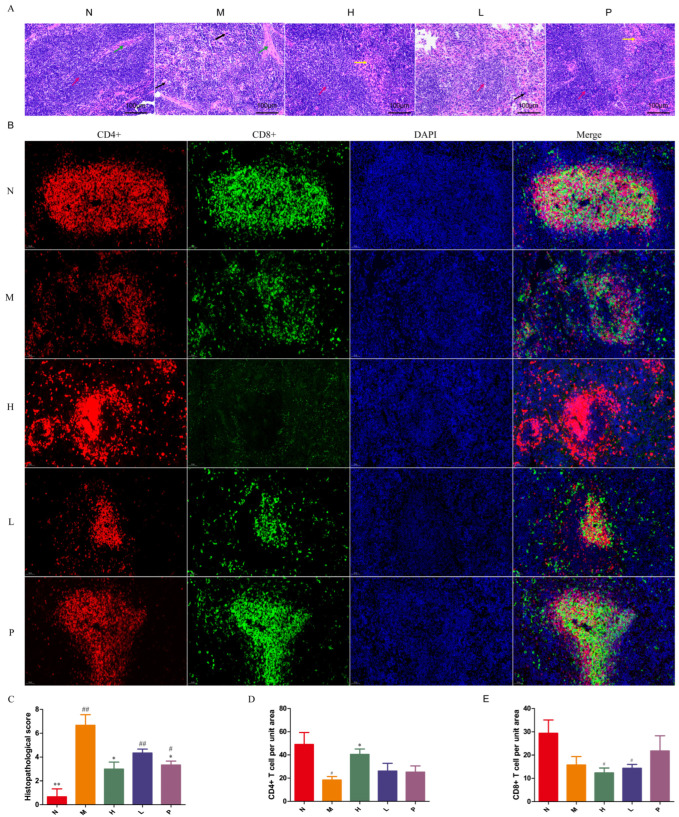
Spleen histopathological and immunofluorescence analysis. (**A**) H&E stain of spleen tissues. The red arrow indicates the white pulp, the green arrow indicates the septa, the yellow arrow indicates the red blood cells, the black arrow indicates the macrophages. (**B**) Immunofluorescence stain of CD4 and CD8 in spleen tissue. The red fluorescence is CD4^+^ cells, the green fluorescence is CD8^+^ cells, the blue fluorescence is the nucleus, and the Merge fluorescence is the overlap of the three kinds of fluorescence. (**C**) Histopathological score; (**D**) CD4^+^ T cell per unit area; (**E**) CD8^+^ T cell per unit area. Data are expressed as the mean ± SD (n = 3). N: normal group; M: immunosuppressed model group; H: DM + high-dose PAOS group; L: DM + low-dose PAOS group; P: DM + levamisole hydrochloride group. ** *p* < 0.01, * *p* < 0.05 vs. M group; ^##^ *p* < 0.01, ^#^ *p* < 0.05 vs. N group.

The spleen serves as a crucial immune organ; dysfunction of the immune system is frequently accompanied by impairment of immune organs. As seen in Figure 3A, the boundary between the white pulp and red pulp in the spleen tissue of normal mice was clear and the morphology of the splenic nodules in the white pulp was regular. However, in the spleen tissue of immunosuppressed mice, the boundary between the white pulp and red pulp was blurred, and some of the splenic nodules were disordered and had irregular shapes, with an increase in macrophages. After treatment with a high dose of PAOSs, the boundary between the red and white pulp in the spleen of immunosuppressed mice became clearer. The findings demonstrated that PAOSs could make the structure of the splenic nodules in the white pulp of immunosuppressed mice change from disordered to regular, and make the demarcation between the red and white pulp clearer. Histopathological evaluation revealed that the model group exhibited severe splenic damage, characterized by the disruption of white pulp structure, marked red pulp expansion, and increased inflammatory cell infiltration, resulting in a significantly elevated histological score (Figure 3C). In contrast, the high-dose group showed near-normal splenic architecture with only mild changes, while the low-dose group exhibited moderate improvement, which was further confirmed by semi-quantitative histological scoring. These results revealed that PAOSs ameliorated CTX-induced splenic lesions in mice.

### 3.5. PAOSs Increased the Percentage of CD4^+^ T Cells in Spleen

To investigate the effect of PAOSs on splenic lymphocytes T cells, the IF was used. CTX significantly reduced the proportion of CD4^+^ cells (Figure 3B–D). Compared with the model group, the fluorescence intensity of CD4^+^ cells in the high-dose group was approximately 2.2-fold higher. However, no remarkable difference was noted in the percentage of CD8^+^ cells between the model group and PAOS group (Figure 3B,E).

**Figure 4:**
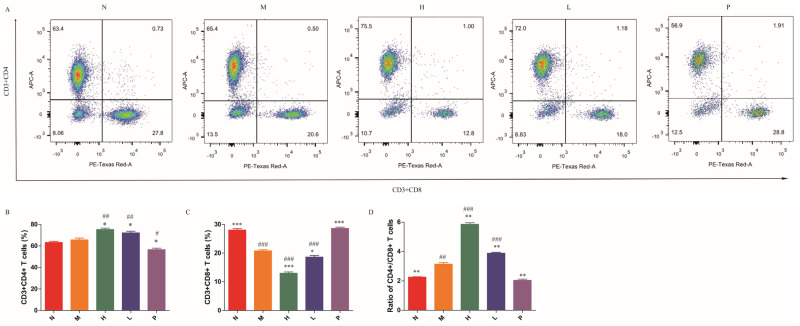
PAOS treatments altered immune cell proportion of CD4^+^ and CD8^+^ T cells in CTX-induced immunosuppression mice. (**A**) The proportion of splenic CD3^+^CD4^+^ and CD3^+^CD8^+^ T cells was detected by flowcytometry; (**B**) the proportion of splenic CD3^+^CD4^+^ T cells; (**C**) the proportion of splenic CD3^+^CD8^+^ T cells; (**D**) the ratio of splenic CD4^+^/CD8^+^ T cells. Data are expressed as the mean ± SD (n = 3). N: normal group; M: immunosuppressed model group; H: DM + high-dose PAOS group; L: DM + low-dose PAOS group; P: DM + levamisole hydrochloride group. *** *p* < 0.001, ** *p* < 0.01, * *p* < 0.05 vs. M group; ^###^ *p* < 0.001, ^##^ *p* < 0.01, ^#^ *p* < 0.05 vs. N group.

The ratios of CD3^+^CD4^+^ or CD3^+^CD8^+^ splenic lymphocytes T cells were explored by flow cytometry. As seen in Figure 4A,C, the proportion of CD3^+^CD8^+^ cells decreased in the PAOS treatment and model group compared to the normal group. Compared with the model group, the percentage of CD3^+^CD4^+^ T cells was significantly elevated by 14,6% in the high-dose PAOS treatment group (Figure 4B). In contrast, CD8^+^ T cell proportions were decreased in the high-dose group. The CD4^+^/CD8^+^ ratio in the high-dose PAOS group was 1.87-fold higher than that in the model group, and was also higher than that in the normal control group, which suggested that PAOSs not only restored immune balance under immunosuppressive conditions but may also promote enhanced CD4^+^ T cell-mediated immune responses (Figure 4B,D). These results suggested that PAOSs boosted the immune function of immunosuppressed mice by raising the percentage of CD4^+^ T cells.

### 3.6. PAOSs Regulated the Serum Metabolic Profiles

**Figure 5:**
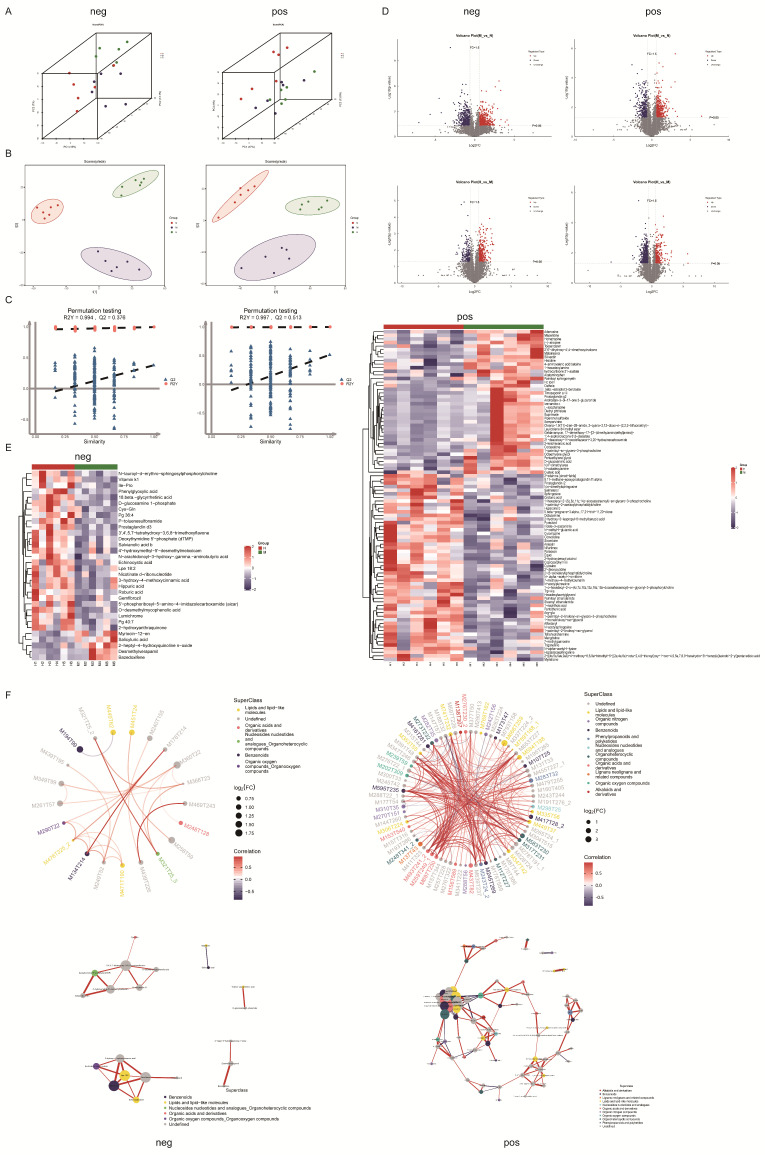
PAOSs regulated serum metabolomic profiles. (**A**) PCA plots of metabolites among N, M, and H groups in negative (left) and positive (right) ion modes; (**B**) OPLS-DA plots of metabolites among N, M, and H groups in negative (left) and positive (right) ion modes; (**C**) permutation testing of OPLS-DA in negative (left) and positive (right) ion modes; (**D**) volcano plots of metabolites of M vs. N groups and H vs. M in negative (left) and positive (right) ion modes, metabolites with VIP values > 1.0, fold change (FC) values > 1.5 or < 0.67, and *p* < 0.05 are colored in red (upregulated) and those with VIP values > 1, FC values < or < 0.67, adjusted *p* < 0.05 in blue (downregulated); (**E**) heatmap analysis between H and M groups in negative (left) and positive (right) ion modes; (**F**) chord diagram (upper) and network diagram (lower) of correlation analysis between significant differential metabolites (VIP > 1, *p* < 0.05) in negative (left) and positive (right) ion modes. N: normal group, M: immunosuppressed model group, H: DM + high-dose PAOS group.

To study the immunoregulatory mechanism of PAOSs in immunosuppressed mice, metabolomic studies of serum were performed in the high-dose PAOS treatment group, model group and normal group. PCA demonstrates the overall distribution trend and sample metabolic differences in all samples. As shown in Figure 5A, the percentages of PC1, PC2, and PC3 were 15%, 13.1% and 7% respectively in negative ion modes, and 17%, 13.8% and 9% respectively in positive ion modes, indicating distinct trends in metabolome separation. OPLS-DA was applied to further illustrate the inter-group differences. Figure 5B shows that there was a distinct separation trend among the NC group, H group and M group (ES+: R2Y = 0.988, Q2 = 0.793; ES−: R2Y = 0.999, Q2 = 0.631), suggesting significant differences in metabolic profiles. Permutation testing indicated that the OPLS-DA model was not overfitted, supporting the reliability of the model (Figure 5C).

Then, metabolites with a variable importance in projection (VIP values > 1.0), fold change (FC values > 1.5 or <0.67), and *p* < 0.05 were identified as significantly differential metabolites. A volcano map shows the differential potential biomarkers between the two groups (M vs. N, H vs. H) in positive and negative ion modes (Figure 5D). The findings of volcano plots showed that the model group had 960 differential metabolites (512 upregulated, 448 downregulated) in the negative ion mode, and owned 677 differential metabolites (358 upregulated, 319 downregulated) in the positive ion mode compared with the normal group. Compared to the model group, the high-dose PAOS group had 490 differential metabolites (317 upregulated, 173 downregulated) in the negative ion mode, and owned 1184 differential metabolites (250 upregulated, 879 downregulated) in the positive ion mode.

To clearly display the expression differences in each potential biomarker, we performed a heatmap analysis (Figure 5E). The heatmap displayed 99 differential metabolites in the positive ion mode, and 33 differential metabolites in the negative ion mode. Compared to the normal group, 2-edahma [dmed-fahfa], 2′-deoxycytidine, coproporphyrin iii, paraoxon, 3′,4′,5,7-tetrahydroxy-3,6,8-trimethoxyflavone, Cys-Gln, Ile-Pro, D-glucosamine 1-phosphate, O-desmethylmycophenolic acid, P-toluenesulfonamide, prostaglandin d3, cytosine, and salvianolic acid b were downregulated remarkably in the model group. Upon administration with PAOSs, the abundance of these metabolites was distinctly elevated. And compared to the model group, the levels of sphingosine, prostaglandin i2, palmitoyl ethanolamide, deoxythymidine 5′-phosohate (dTMP) and 2-deoxycytidine were significantly increased, and the levels of prostaglandin g2, adenosine, and cysteic acid were significantly reduced in the high-dose PAOS treatment group.

To further explore the interrelationships of metabolites under changing biological conditions, correlation analysis between significant differential metabolites (VIP > 1, *p* < 0.05) was performed. Different metabolites exhibit synergistic or mutually exclusive relationships. Both the chord diagram and network diagram displayed metabolite pairs with a correlation coefficient |r| > 0.8 and *p* < 0.05 (Figure 5F), which demonstrated the correlations between various metabolites. The results showed the correlations between differential metabolites in the positive (right) and negative (left) ion mode. In the chord diagram (upper), the starting points of the inner circle links represent significant differential metabolites; the size of the points corresponds to the log_2_FC value of the respective differential metabolites; different colors on the outer circle indicate the categories to which the significant differential metabolites belong; and the connecting lines represent the correlations between the corresponding metabolites. The shade of the color is related to the absolute correlation coefficient, with red denoting positive associations and blue denoting negative associations. In the network diagram (lower), the points represent significant differential metabolites; the size of the points correlates with the connectivity degree; and the line width reflects the magnitude of the correlation coefficient, with thicker lines indicating stronger correlations.

**Figure 6:**
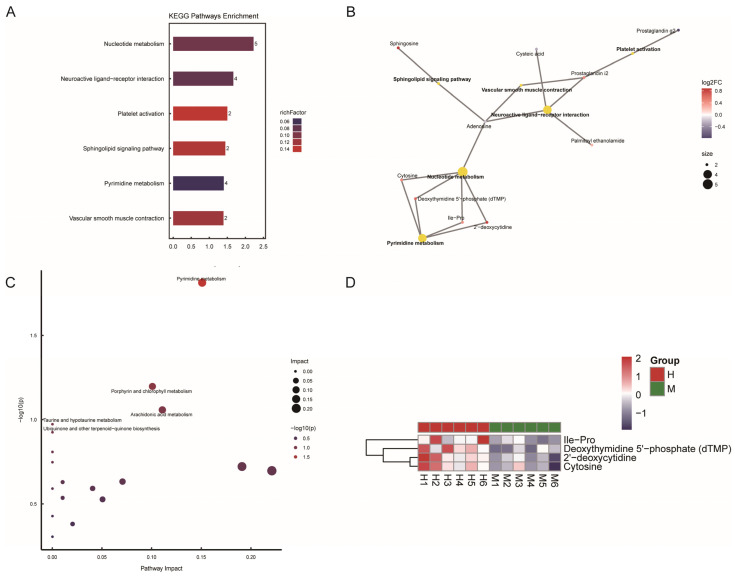
The effects of PAOSs on metabolic pathways in serum. (**A**) KEGG pathway enrichment analysis; (**B**) KEGG pathway network diagram; (**C**) pathway topology analysis; (**D**) heatmap analysis of pyrimidine metabolism-associated differential metabolites.

To investigate the potential mechanism of PAOSs on immunosuppression, KEGG analysis was performed to explore significant functional pathways. Differential metabolites with statistical significance were primarily associated with nucleotide metabolism, neuroactive ligand–receptor interaction, platelet activation, the sphingolipid signaling pathway, pyrimidine metabolism and vascular smooth muscle contraction (Figure 6A). The KEGG pathway network diagram (Figure 6B) better illustrates the connections between various differential metabolites and pathways. Pathway topology analysis (Figure 6C) emphasized metabolic pathways and identified key pathways highly correlated with metabolite differences, comprising pathways related to pyrimidine metabolism, porphyrin and chlorophyll metabolism, arachidonic acid metabolism, taurine and hypotaurine metabolism, as well as ubiquinone and other terpenoid–quinone biosynthesis. Collectively, KEGG enrichment analysis and pathway topology analysis identified pyrimidine metabolism as the key pathway that mediated the immunostimulatory activity of PAOSs. To further investigate the expression profiles of differential metabolites related to the pyrimidine pathway, heatmap visualization was performed. Figure 6D shows that the levels of Ile-Pro, dTMP, 2′-deoxycytidine, and cytosine were elevated in the high-dose PAOS treatment group, compared to the model group. We present a KEGG pathway mapper of the differential metabolic pathway—the pyrimidine metabolic pathway—wherein the red dots represent upregulated differential metabolites (Figure S1). These findings suggested that PAOSs exerted an immunostimulatory effect by increasing the levels of Ile-Pro, dTMP, 2′-deoxycytidine, and cytosine, thus driving the upregulation of pyrimidine metabolism.

### 3.7. Regulatory Effects of PAOSs on SCFA-Producing Bacteria and SCFA Levels

**Figure 7:**
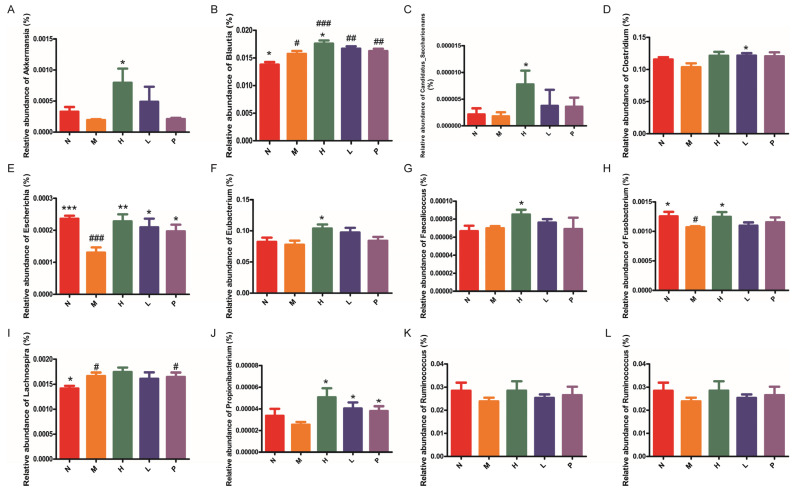
The relative abundance of SCFA-producing bacteria at genus levels after PAOS intervention. The relative abundance of *Akkermansia* (**A**), *Blautia* (**B**), *Candidatus_Saccharicenans* (**C**), *Clostridium* (**D**), *Escherichia* (**E**), *Eubacterium* (**F**), *Faecalicoccus* (**G**), *Fusobacterium* (**H**), *Lachnospira* (**I**), *Propionibacterium* (**J**), *Ruminococcus* (**K**) and *Xylanimicrobium* (**L**). Data are expressed as the mean ± SD (n = 6). N: normal group; M: immunosuppressed model group; H: DM + high-dose PAOS group; L: DM + low-dose PAOS group; P: DM + levamisole hydrochloride group. *** *p* < 0.001, ** *p* < 0.01, * *p* < 0.05 vs. M group; ^###^ *p* < 0.001, ^##^ *p* < 0.01, ^#^ *p* < 0.05 vs. N group.

**Figure 8:**
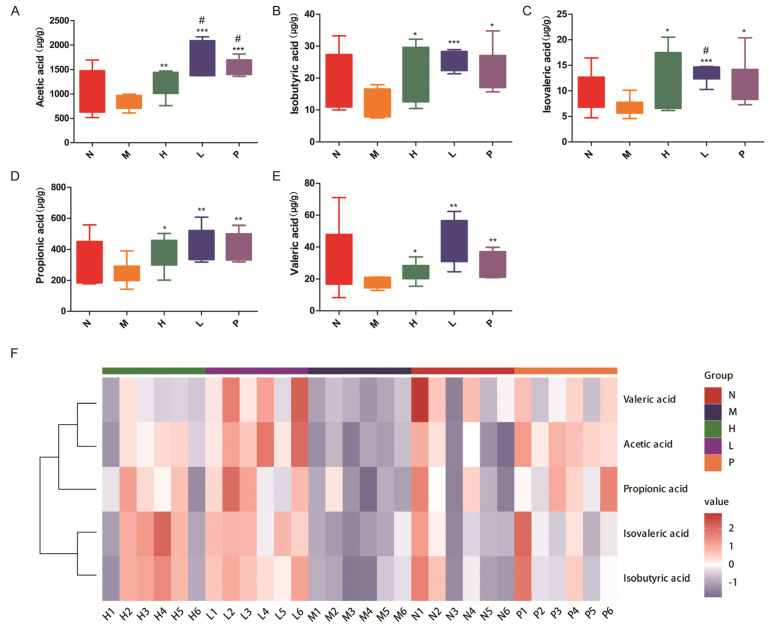

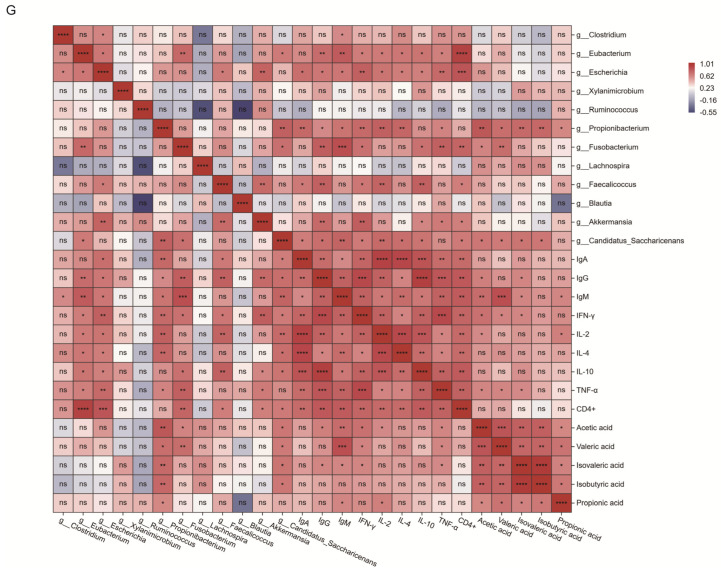
Effects of PAOSs on SCFAs in the gut and Spearman correlation analysis. The levels of acetic acid (**A**), isobutyric acid (**B**), isovaleric acid (**C**), propionic acid (**D**), and valeric acid (**E**); (**F**) the heatmap of SCFAs. N: normal group; M: immunosuppressed model group; H: DM + high-dose PAOS group; L: DM + low-dose PAOS group; P: DM + levamisole hydrochloride group. *** *p* < 0.001, ** *p* < 0.01, * *p* < 0.05 vs. M group, ^#^ *p* < 0.05 vs. N group. (**G**) Spearman correlation analysis among the microbiota, SCFAs, immunoglobulins and cytokines indicators, and percentage of CD4^+^ T cells. The red and blue colors denote positive and negative correlations, respectively, and the intensity of the color is proportional to the strength of Spearman correlation. Data are expressed as the mean ± SD (n = 6). ns, not significant; *p* ≥ 0.05, **** *p* < 0.0001, *** *p* < 0.001, ** *p* < 0.01, * *p* < 0.05.

SCFA-producing bacteria are the core functional group of the gut microbiota, and their SCFA products are essential for maintaining intestinal barrier integrity, regulating immunity, and mediating host metabolism. PAOS treatment induced modest alterations in the gut microbial profile, with specific changes observed in bacteria associated with SCFA production. Based on their functional relevance, subsequent analyses focused on representative SCFA-producing bacteria. To clarify the impact effects of PAOSs on functional gut microbiota and their metabolites, the relative abundance of intestinal SCFA-producing bacteria at genus levels and the levels of SCFAs were explored. As presented in Figure 7A–L, in comparison with the model group, the relative abundance of SCFA-producing bacteria at genus levels, including *Akkermansia, Blautia, Candidatus_Saccharicenans, Escherichia, Eubacterium, Faecalicoccus, Fusobacterium,* and *Propionibacterium*, showed a pronounced increase (*p* = 0.025, *p* = 0.0452, *p* = 0.0448, *p* = 0.0079, *p* = 0.0276, *p* = 0.0248, *p* = 0.0440, *p* = 0.0201, respectively) in the high-dose PAOS treatment group. And the relative abundance of *Clostridium, Lachnospira, Ruminococcus* and *Xylanimicrobium* were elevated in the PAOS treatment group, compared with the model group. As shown in Figure 8A, the concentrations of acetic acid in the high-dose, low-dose PAOS, and positive control groups were 1.51, 2.07, and 1.87-fold higher than those in the model group, respectively (*p* = 0.0077, *p* = 0.0002, *p* < 0.0001). Compared with the model group, the concentrations of isobutyric acid in the high-dose, low-dose PAOS, and positive control groups were increased by 1.82, 1.99, and 1.71-fold, respectively (Figure 8B, *p* = 0.0274, *p* = 0.001, *p* = 0.0226). Compared with the model group, isovaleric acid concentrations were significantly increased by 1.97, 2.02, and 1.70-fold in the high-dose, low-dose PAOS, and positive control groups, respectively (Figure 8C, *p* = 0.0233, *p* < 0.0001, *p* = 0.0445). As shown in Figure 8D, the concentrations of propionic acid in the high-dose, low-dose PAOS, and positive control groups were 1.50, 1.75, and 1.65-fold higher than those in the model group, respectively (*p* = 0.0411, *p* = 0.0066, *p* = 0.0086). And the level of valeric acid was increased by 1.4, 2.46, and 1.77-fold in the high-dose, low-dose PAOS, and positive control groups, respectively (Figure 8E, *p* = 0.0345, *p* = 0.002, *p* = 0.0045). The heatmap of SCFAs (including acetic acid, isobutyric acid, isovaleric acid, propionic acid, and valeric acid) is shown in Figure 8F. These findings indicated that PAOSs exert immunity effects by elevating the composition of intestinal SCFA-producing bacteria and the levels of SCFAs.

Additionally, Spearman’s rank correlation was explored to assess the associations among microbiota, immunoglobulins and cytokines, and SCFAs (Figure 8G). The results showed a positive correlation of *Escherichia* with immunoglobulins and cytokines, and CD4^+^; a positive correlation of *Propionibacterium* with immunoglobulins, cytokines, and SCFAs; a positive correlation of *Fusobacterium* with immunoglobulins (IgM, IgG), cytokines (IL-4, IL-10, TNF-α, INF-γ), valeric acid, acetic acid, and CD4^+^; a positive correlation of *Akkermansia* with IgG, INF-γ, IL-10, TNF-α, and CD4^+^; a positive correlation of *Candidatus_Saccharicenans* with immunoglobulins and cytokines (IL-4, IL-10, IL-2, INF-γ), SCFAs, and CD4^+^. The percentage of CD4^+^ cells was positively correlated with immunoglobulins and cytokines. The levels of immunoglobulins and cytokines were positively correlated with acetic acid, propionic acid, isovaleric acid and valeric acid. These results suggested that PAOSs enhanced immunity by elevating the level of SCFA-producing bacteria and SCFAs.

## 4. Discussion

CTX is a commonly used alkylating agent-type immunosuppressant in clinical practice and experimental research, which exerts cytotoxic effects by damaging the DNA of proliferatively active cells (particularly through DNA cross-linking) [31]. Various natural substances possess the capacity to repair immune system damage, owing to their wide-ranging biological effects, with no detectable side effects [15]. For example, the functional oligosaccharides have immune enhancement effects, such as mannan oligosaccharides (MOS) [32], oligosaccharides from the seeds of Dolichos lablab L. [33], and konjac glucomannan oligosaccharides [34]. In this study, we demonstrated that PAOSs improved immune responses in immunocompromised mice by stimulating the secretion of immunoglobulins and cytokines, elevating the percentage of CD4^+^ cells in the spleen, suppressing oxidative stress, regulating serum metabolomic profiles, and modulating SCFA-producing bacteria and their metabolite production.

The immunoglobulins, cytokines and CD4^+^ T cells are essential for regulating immune responses [13]. Immunoglobulins are secreted by B cells, and are key elements of the humoral immune response; for example, IgA, IgG, and IgM activate the complement cascade through the formation of antibody–antigen complexes [35,36]. IgG is the most abundant type of immunoglobulin and mediates natural passive immunity, including antibacterial, antiviral, and antitoxic effects. IgA exhibits bacteriolytic activity and plays a role in promoting phagocytosis and aggregation in the body [37]. IgM is the first antibody to appear in humoral immunity, and the detection of serum IgM levels serves as an indicator for the early diagnosis of infections [38]. Curdlan oligosaccharides (GOSs) increased the secretion of Igs (IgG by 50.6–74.7%, IgA by 31.3–34.9%, IgM by 28.3–66.7%) in immunosuppressed mice [39]. PAOSs elevated the levels of IgG and IgM, which suggested that PAOSs enhanced immunity by promoting B cell-mediated humoral immunity. CD4^+^ T cells, a core subset of T lymphocytes, function as central orchestrators of adaptive immune responses with multiple pivotal roles, primarily mediated by their differentiation into specialized subpopulations (Th1, Th2, Th17 and Tregs cells) and cytokine secretion [13]. IL-2, IFN-γ, and TNF-α secreted by Th1 cells are key factors in mediating cell-mediated immune responses. Th2 cells produce IL-4 and IL-10 that can promote humoral or allergic immune responses by promoting B cell proliferation and antibody production [21]. IL-2 and IFN-γ modulate immune activity by promoting the proliferation of T and B lymphocytes and inducing the differentiation of regulatory Th1 cells [40,41]. TNF-α induces the recruitment of immune cells and promotes the activation of immune cells (macrophages and T cells) [42]. IL-4 and IL-10 are the core regulators of humoral immunity, promoting the proliferation of B cells and the production of corresponding antibodies [43]. These cytokines primarily play a crucial role in facilitating communication between cells in the immune system and participate in restoring homeostasis by coordinating immune cells [44]. Therefore, the secretion of cytokines mediated by immune cells modulates immune function. Earlier studies have shown that GOSs stimulate the release of IL-1β, IL-6 and TNF-α to reduce CTX-induced immune damage [39]. *C. pilosula* oligosaccharides elevate immunoglobulin contents, stimulate the proliferation of splenic lymphocytes, and promote the production of IL-2 and IFN-γ to enhance immune effects [45]. Consistent with these results, PAOSs elevated the level of IL-2, IFN-γ, TNF-α and IL-10. These findings indicated that PAOSs regulated immunity by stimulating the production of CD4^+^ cells and promoting the secretion of cytokines.

High doses of CTX triggers an overproduction of reactive oxygen species, leading to oxidative stress [46]. Lipid peroxidation caused by oxidative stress damages cell membranes, weakening immunity and altering the T cell receptor complex [47,48]. MDA levels are widely used to assess oxidative stress. The critical antioxidant enzymes (SOD, CAT, and GSH-Px) are directly involved in the detoxification of free radicals [49]. PAOSs increased the activity of antioxidant enzymes (SOD, GSH-Px, and CAT) and decreased the level of MDAs. These results suggested that PAOSs enhanced immunity by reducing oxidative stress.

As indicators of cellular function, metabolites have been validated to reflect aberrant alterations in immune responses [50]. Our results showed that 13 metabolomics, including 2-edahma [dmed-fahfa], 2′-deoxycytidine, coproporphyrin iii, paraoxon, 3′,4′,5,7-tetrahydroxy-3,6,8-trimethoxyflavone, Cys-Gln, Ile-Pro, D-glucosamine 1-phosphate, O-desmethylmycophenolic acid, P-toluenesulfonamide, prostaglandin d3, cytosine, and salvianolic acid b, were reduced in immunosuppressive mice. After PAOS intervention, the levels of these metabolites were significantly elevated. Deoxycytidine is phosphorylated by dCK to form dCTP, and dCTP pyrophosphatase 1 maintains dNTP pool homeostasis by hydrolyzing dCTP; the accumulation of dCTP leads to DNA replication errors, which impair immune cell proliferation and function, thereby revealing the core association between pyrimidine metabolism and immune cell homeostasis [51]. 3′,4′,5,7-trihydroxy-6,8,3-trimethoxyflavon regulates immune function by increasing IgE, IgG1, IL-4 and IL-10 production, and improving the antigen presentation capability in bone marrow-derived dendritic cells [52]. Supplementation with glutamine dipeptide (primarily Gly-Gln, glycyl–glutamine) improves the recovery of postoperative immune function and significantly reduces the infection rate in critically ill surgical patients. Diacyl α-D-glucosamine 1-phosphate is a B lymphocyte mitogen that can directly activate B cell proliferation. It also functions as an essential precursor in the synthesis of lipopolysaccharide and participates in innate immune recognition [53]. Salvianolic acid B has been reported to be associated with immunomodulatory effects, including potential influences on immune organ indices, T cell balance, and cytokine production [54]. Cytosine enhances innate and adaptive immune responses by promoting dendritic cell maturation, B cell activation, and the secretion of Th1-type cytokines (e.g., IL-12 and IFN-γ) [55]. And in contrast to the model group, the levels of sphingosine, prostaglandin i2, and dTMP were significantly increased in the PAOS treatment group. Sphingolipid metabolism is implicated in multiple physiological processes, encompassing cell proliferation, inflammation, and immune responses [56]. Prostaglandin-related metabolites have been reported to participate in immune regulation, including potential effects on T cell differentiation and cytokine production [57]; however, their roles are complex and context-dependent, and their significance in this study remains to be further clarified. Studies have reported that the reprogramming of pyrimidine metabolism acts as the primary foundation for the rapid proliferation of T cells, and that dTMP, as a direct precursor for DNA synthesis, requires adequate supply to act as an indispensable prerequisite for the clonal expansion of T cells [58]. Metabolite identification in this study was based on database matching and remains putative due to the lack of MS/MS validation; further validation is required to confirm their identities and functions. Taken together, these metabolites might be involved in the initiation of immune responses. The findings implied that PAOSs enhanced immunity by regulating the serum metabolic profiles.

De novo pyrimidine synthesis serves as a rate-limiting step for T cell proliferation and effector function [59], and pyrimidine metabolism acts as a key driver for the production of T cell effector molecules [60]. Activation of the pyrimidine metabolism pathway enhances both innate and adaptive immune responses by maintaining nucleotide homeostasis, promoting immune cell proliferation and activation, upregulating antigen presentation efficiency, and regulating the release of inflammatory factors [61,62]. Increasing evidence suggests that immune activation is accompanied by metabolic reprogramming, during which nucleotide biosynthesis pathways are upregulated to support rapid cell division and effector functions [63]. In this study, both KEGG analysis and pathway topology analysis revealed that pyrimidine metabolism was a key pathway mediating the immunostimulatory effect of PAOSs, and that key differential metabolites, including Ile-Pro, dTMP, 2′-deoxycytidine, and cytosine, were involved in pyrimidine metabolism. PAOS treatment was associated with enhanced CD4^+^ T cell responses, elevated cytokine production, and increased immunoglobulin levels, indicating an overall activation of adaptive immunity. The observed upregulation of pyrimidine-related metabolites may therefore reflect increased nucleotide demand during lymphocyte activation and proliferation. In addition, the reduction in oxidative stress observed in PAOS-treated groups may contribute to a favorable intracellular environment that supports metabolic activity and immune cell function [64]. Collectively, these findings suggest that PAOSs may enhance immune responses, at least in part through metabolic reprogramming involving pyrimidine metabolism. However, further studies are required to confirm the causal relationship between pyrimidine metabolism and immune regulation.

CTX can disrupt gut microbiota balance, leading to gut dysbiosis, thereby further affecting intestinal immune homeostasis [65,66]. Among gut microbes, SCFA-producing bacteria play a critical role in maintaining host physiological functions. These bacteria contribute to intestinal and systemic immune regulation primarily through the production of SCFAs, which act as key signaling metabolites [67]. Emerging evidence indicates that SCFAs, including acetate, propionate, and butyrate, are involved in modulating immune responses, oxidative stress, and metabolic homeostasis [28,68]. Therefore, changes in SCFA-producing bacteria may represent an important functional link between gut microbial alterations and host immune regulation in the present study. Multiple polysaccharides and oligosaccharides have been proven to exhibit the effects of regulating gut microbial function and enhancing immune function [21]. For example, Atractylodes lancea rhizome polysaccharide increased the abundance of *Lactobacillus* and *Faecalibaculum*, which reversed the reduction in SCFAs [69]. Holothuria leucospilota polysaccharides increased the abundance of SCFA-producing bacteria (*Lactobacillus, Prevotellaceae_UCG-001, Akkermansia*) and the SCFA concentration [13]. Prebiotic xylo-oligosaccharides increases the abundance of *Lactobacillus*, which enhances SCFA (lactate and acetate) production [70]. This study showed that PAOSs raised the abundance of SCFA-producing genera, including *Akkermansia, Blautia, Candidatus_Saccharicenans, Escherichia, Eubacterium, Faecalicoccus, Fusobacterium,* and *Propionibacterium*. Alterations in *Escherichia* and *Fusobacterium* were observed following PAOS treatment; however, their biological roles are context-dependent and may differ across species, strains, and host conditions [71]. Thus, their functional significance in this study remains uncertain and warrants further investigation. PAOSs increased SCFA concentrations, including acetic acid, isobutyric acid, isovaleric acid, propionic acid, and valeric acid. The Spearman correlation analysis indicated that PAOSs increased SCFA levels by boosting the relative abundance of SCFA-producing microbes, then stimulating the release of cytokines and immunoglobulins.

This study has several limitations that should be acknowledged. The microbiome analysis was primarily focused on SCFA-producing bacteria, and a comprehensive characterization of the overall gut microbial community was not performed. In addition, the associations between gut microbiota, metabolites, and immune responses observed in this study do not necessarily indicate causality, and the underlying mechanisms require further investigation.

## 5. Conclusions

In conclusion, PAOSs exerted significant immunomodulatory effects by enhancing the release of immunoglobulins and cytokines, elevating the proportion of CD4^+^ cells in the spleen, alleviating oxidative stress, regulating the serum metabolic profiles and upregulating pyrimidine metabolism. In addition, PAOSs specifically modulated SCFA-producing bacteria, leading to increased SCFA production. Collectively, these findings indicated that PAOSs enhance immune function through the coordinated regulation of host metabolism and the SCFA-producing gut microbiota axis, providing mechanistic insight into the immunomodulatory activity of insect-derived oligosaccharides and supporting their potential as bioactive agents for immune-related regulation.

## Figures and Tables

**Table 1:** Histological scoring system.

Score	White Pulp Integrity	Red Pulp Expansion	Inflammatory Cell Infiltration
0	Normal structure with clear boundaries	Normal distribution	No obvious inflammatory cells
1	Slight reduction or blurred boundary	Mild expansion	Mild infiltration
2	Moderate reduction and partial disorganization	Moderate expansion	Moderate infiltration
3	Severe disruption of structure	Marked expansion with congestion	Marked infiltration
4	Nearly absent white pulp	Severe expansion with hemorrhage	Severe and diffuse infiltration

## Data Availability

The original contributions presented in this study are included in the article/Supplementary Material. Further inquiries can be directed to the corresponding authors.

## Supplementary Materials

The following supporting information can be downloaded at: https://www.mdpi.com/article/10.3390/biom16040496/s1, Figure S1: KEGG pathway mapper of the pyrimidine pathway.

## Abbreviations

The following abbreviations are used in this manuscript:

PAOS	*Periplaneta americana* oligosaccharides
CTX	cyclophosphamide
SCFA	short-chain fatty acids
Igs	immunoglobulins
SOD	superoxide dismutase
MDA	malondialdehyde
CAT	catalase
GSH-Px	glutathione peroxidase
H&E	hematoxylin and eosin
GC	gas chromatography
PCA	principal component analysis
OPLS-DA	orthogonal least squares discriminant analysis
dTMP	deoxythymidine 5′-phosohate
MOS	mannan-oligosaccharides
GOS	curdlan oligosaccharides
