# Supplementary figures and images for: Immunomodulatory Effects of Periplaneta americana Oligosaccharides Through SCFA-Producing Gut Microbiota and Metabolic Regulation in Immunosuppressed Mice
Source: Biomolecules. 2026 Mar 25;16(4):496. doi: 10.3390/biom16040496 (PMC13113876; doi:10.3390/biom16040496)

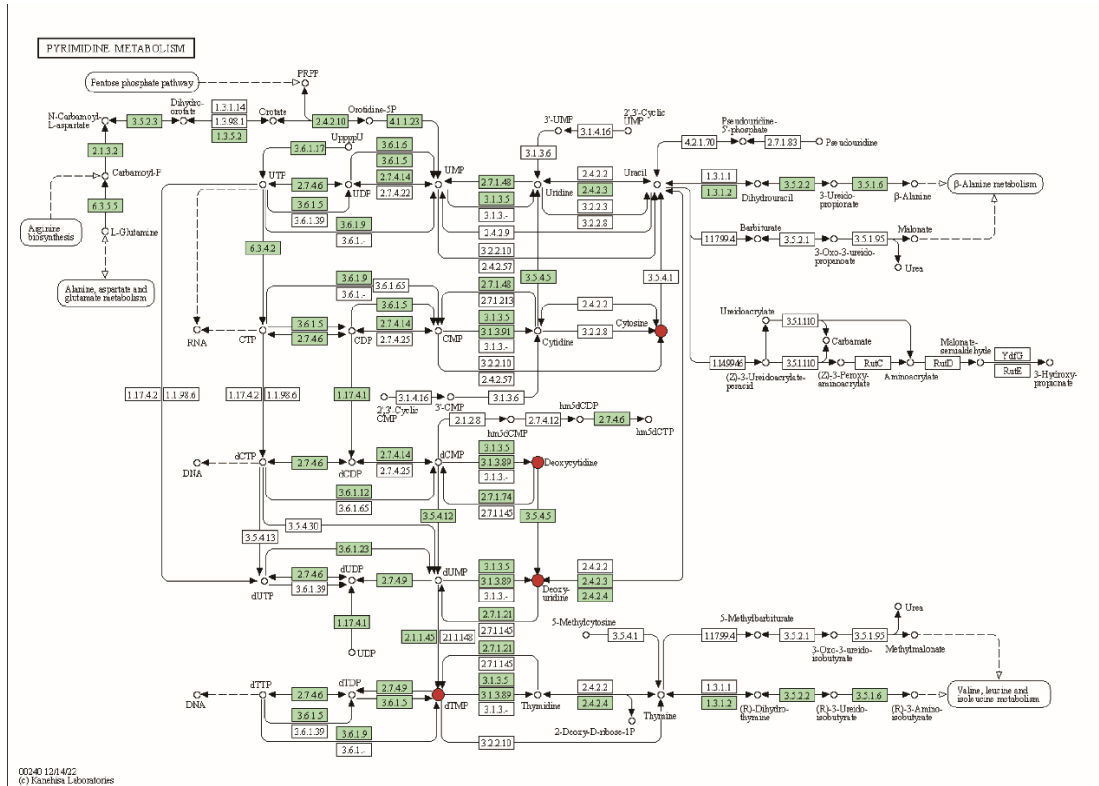

Figure S1. KEGG pathway mapper of the pyrimidine pathway.

Supplement: Supplementary file 1 [file biomolecules-16-00496-s001.zip › biomolecules-4168263-supplementary.pdf]
